# Sterically Facilitated Intramolecular Nucleophilic NMe_2_ Group Substitution in the Synthesis of Fused Isoxazoles: Theoretical Study

**DOI:** 10.3390/molecules25245977

**Published:** 2020-12-17

**Authors:** Alexander S. Antonov, Elena Yu Tupikina, Valerii V. Karpov, Valeriia V. Mulloyarova, Victor G. Bardakov

**Affiliations:** Institute of Chemistry, St. Petersburg State University, Universitetskii pr. 26, 198504 St. Petersburg, Russia; e.tupikina@spbu.ru (E.Y.T.); st055697@student.spbu.ru (V.V.K.); st057550@student.spbu.ru (V.V.M.); st081032@student.spbu.ru (V.G.B.)

**Keywords:** nucleophilic substitution, heterocyclization, isoxazole, quantum chemical calculations

## Abstract

The influence of steric repulsion between the NMe_2_ group and a second *ortho*-(*peri*-)substituent in the series of 1-dimethylaminonaphthalene and *N*,*N*-dimethylanilene *ortho*-oximes on the ease of the NMe_2_ group’s intramolecular nucleophilic substitution is studied. Possible reaction intermediates for three mechanisms are calculated (ωB97xd/def-2-TZVP), and their free Gibbs energies are compared to model reaction profiles. Supporting experiments have proved the absence of studied reactivity in the case of simple 2-dimethylaminobenzaldoxime, which allowed us to establish reactivity limits. The significant facilitation of NMe_2_ group displacement in the presence of bulky substituents is demonstrated. The possibility of fused isoxazoles synthesis via the intramolecular nucleophilic substitution of a protonated NMe_2_ group in the aniline and naphthalene series is predicted.

## 1. Introduction

**Scheme 1:**
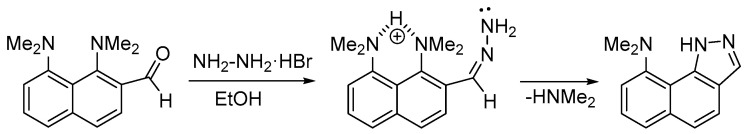
Substitution of the NMe_2_ group promoted by acidic catalysis.

**Scheme 2:**
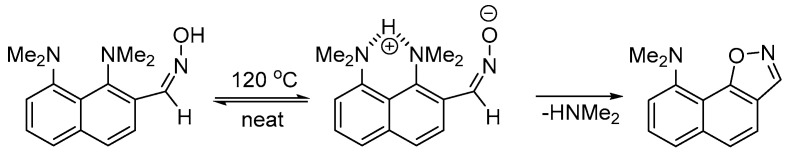
Tentative mechanism of NMe_2_ group substitution in *ortho*-oximes of DMAN.

The dimethylamino group is generally considered as one of the worst leaving groups in organic chemistry. That is why there are only a few examples of its substitution in aromatic substrates. Known cases mostly involve the activation by quaternization of an NMe_2_ group [1,2,3,4], or the introduction of strong electron withdrawing groups in the aromatic core [5,6,7,8,9]. The substitution of an unactivated NMe_2_ group is much less known and occurs under metal [10,11] or acidic catalysis [12]. The latter, published in 2011, was demonstrated for the *ortho*-substituted 1,8-bis(dimethylamino)naphthalenes (hydrazones, imines and oximes), which in acidic media undergo intramolecular nucleophilic NMe_2_ group substitution, leading to the formation of the following nitrogen heterocycles: benzo[*g*]indazoles, benzo[*g*]quinazolines, and naphtho[2,1-*d*]isoxazoles (Scheme 1) [12]. It was shown that, unlike hydrazones, oximes do not require an external acidic catalyst to initiate the reaction. The authors attributed this phenomenon to the high basicity of 1,8-bis(dimethylamino)naphthalene (DMAN; p*K*_a_ = 12.1 in H_2_O [13], 18.62 in MeCN [14], 7.5 in DMSO [15]), which facilitated the proton transfer from the oxime to the DMAN moiety, thus providing the necessary acidic catalysis (Scheme 2). Indeed, much less basic *N*,*N*-dimethylaniline (DMA) derivatives (p*K*_a_ of DMA = 5.1 in H_2_O [16]) showed no reactivity under similar conditions.

In recent years, it was demonstrated that the unique reactivity features of 1,8-bis(dimethylamino)naphthalene derivatives originate from the strong repulsion of NMe_2_ groups and their stable *in*,*in*-conformation. Thus, *ortho*-alkynyl-DMANs undergo a palladium-catalyzed transformation into less strained benzo[*g*]indoles [17]. The treatment of *ortho*-trifluoromethyl-DMAN with organolithiums also leads to the loss of a steric strain via the formation of dihydrobenzo[*g*]indoles [18,19]; the *ortho*-ketimines of DMAN react with nitriles in the presence of a strong base, giving less strained benzo[*h*]quinazolines [11]. However, none of the mentioned reactions require the high basicity feature of DMAN.

In this work, through means of quantum–chemical calculations, we aimed to answer the question of whether steric repulsion is a reason for the surprisingly easy substitution of the NMe_2_ group in DMAN *ortho*-oximes, if it is possible to facilitate NMe_2_ group displacement in *N*,*N*-dimethylaniline derivatives via the introduction of a bulky substituent to the *ortho*-position, and if this approach could serve as a method for the synthesis of fused isoxazoles—an important class of five-membered aromatic heterocycles, which molecules are present as a pharmacophore essential for the biological activity in many drugs and bioactive natural products [20].

## 2. Results

It is widely known that aromatic nucleophilic substitution can be described by three possible mechanisms: addition–elimination S_N_Ar(AE), elimination–addition S_N_Ar(EA) and benzyne S_N_Ar(B). The latter requires hydrogen in the *ortho*-position of the leaving group, which is not the case here, so this mechanism was out of consideration. Keeping in mind that S_N_Ar(EA) is commonly associated with diazonium salts, when the elimination of N_2_ leads to the formation of aromatic carbocations, we mainly focused on the S_N_Ar(AE) mechanism in our calculations.

**Scheme 3:**
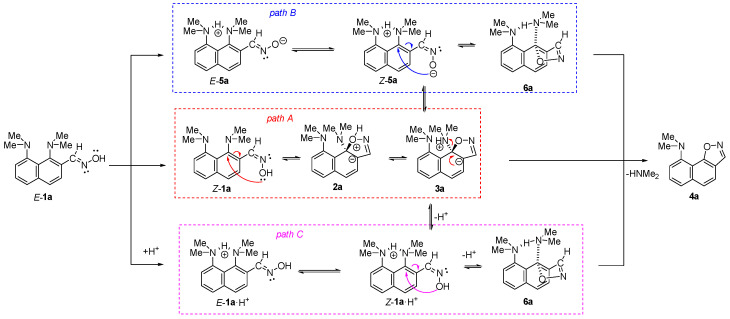
Proposed pathways for the S_N_Ar(AE) mechanism of **1a** → **4a** transformation.

We started our work with the search for the most optimal pathway of the transformation of proton sponge aldoxime **1a** into isoxazole **4a**. Since aldoxime **1a** exists solely as a *E*-isomer, [12] which is not preorganized for intramolecular nucleophilic attack, the reaction mechanism should include the *E*→*Z* isomerization step. Keeping the possibility of proton transfer from oxime to the proton sponge moiety in mind, it is possible to suggest “neutral” (A), “zwitterionic” (B) and “acidic” (C) reasonable reaction pathways (Scheme 3).

“Neutral” pathway A starts with the *E*→*Z* isomerization of **1a**, followed by an intramolecular nucleophilic attack of oxygen on C-NMe_2_ carbon and the formation of **2a**. The further proton transfer from isoxazole oxygen to the NMe_2_ group facilitates the displacement of the latter in **3a**.

The second “zwitterionic” pathway B is initiated by a proton transfer from the OH group to the NMe_2_ group, with the formation of zwitterion *E*-**5a**. The isomerization of *E*-**5a** into *Z*-**5a** allows an intramolecular attack with the formation of **3a** and finally **4a** after displacement of dimethylamine. Alternatively, the isoxazole ring closure and displacement of the NMe_2_ group in *Z*-**5a** could occur as a concert process via intermediate **6a**.

The third “acidic” path C starts with the dissociation of the OH bond (*pK_a_* of oximes is generally ≈20 in DMSO [21]), thus providing a catalytic amount of protons. Being released to the media, these protons are abstracted by oxime *E*-**1a** to form *E*-**1a**·H^+^. Further *E*→*Z* isomerization is followed by the nucleophilic attack and displacement of the NMe_2_ group, either as a step-by-step (via **3a**) or concert (via **6a**) process.

**Figure 1:**
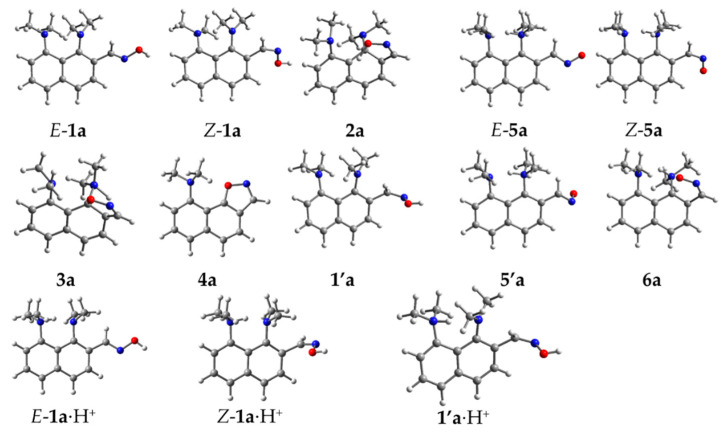
Optimized geometries of studied reaction intermediates for **1a** → **4a** transformation.

**Scheme 4:**
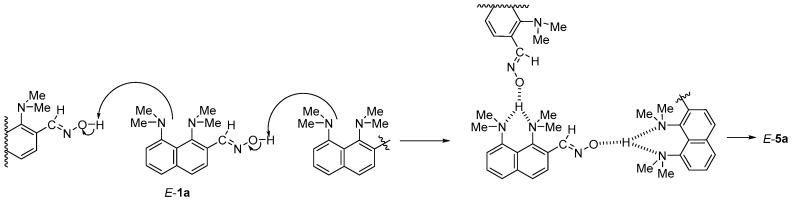
Possible means of proton transfer upon transformation *E*-**1a** → *E*-**5a**.

**Scheme 5:**
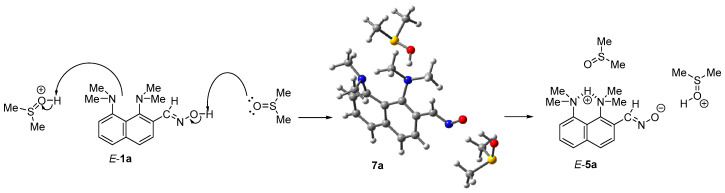
Simplified means of proton transfer upon transformation *E*-**1a** → *E*-**5a**. Equilibrium geometry of transition state **7a** is shown.

For all intermediate forms of the three mechanisms, we calculated equilibrium geometries and harmonic vibrational frequencies. Based on IR frequencies, it was found that the structures *E*-**1a**, *E*-**1a**·H^+^, *Z*-**1a**, *Z*-**1a**·H^+^, *E*-**5a**, *Z*-**5a** and **4a** correspond to the local minima, while the structures **2a**, **3a**, and **6a**·H^+^ are saddle points (i.e., transition states). The transfers *E*-**1a** → *Z*-**1a** and *E*-**1a**·H^+^ → *Z*-**1a**·H^+^ occur through the transition states, which are labeled as **1’a** and **1’a**·H^+^, respectively; the transition states located between *E*-**5a** and *Z*-**5a**, and *E*-**1a** and *E*-**5a** are labeled as **5’a** and **7a,** respectively (Figure 1). Since the studied reaction is carried out without any solvent (in neat melt of **1a**) or in aprotic DMSO, the transition of *E*-**1a** → *E*-**5a** is bound to be a concert process of a proton transfer from one oxime molecule to another one (Scheme 4). However, the calculation of the transition state for this transformation (**7a**)—even in the form of cyclic trimer—is computationally costly. In order to reduce the calculation time, we performed the modeling of the proton transfer in a simplified form using a DMSOH^+^ molecule as a proton donor for the dimethylamino group and a DMSO molecule as a proton acceptor for the C(H)NOH moiety (Scheme 5).

**Scheme 6:**
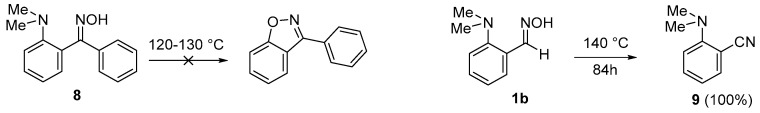
Thermolysis of DMA-based oximes **1b** and **8**.

**Figure 2:**
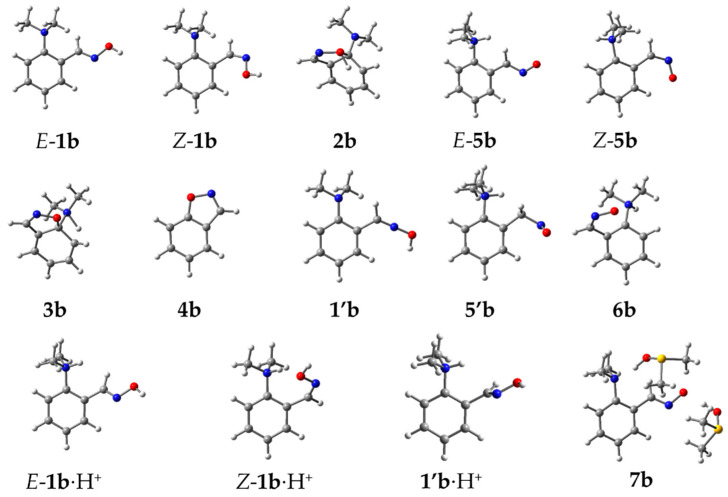
Optimized geometries of studied reaction intermediates for **1b** → **4b** transformation.

As was briefly mentioned in the introduction, unlike proton sponge-based aldoximes and ketoximes, 2-(dimethylamino)benzophenone oxime **8** under prolonged heating at 120–130 °C remains unchanged (Scheme 6) [12]. In the present work, we performed an additional experiment to ensure that the similar DMA-based aldoxime **1b** undergoes no NMe_2_ group substitution under the selected conditions. Indeed, after heating at 140 °C for 84 h, **1b** quantitively converts into nitrile **9** (according to NMR spectra of the reaction mixture), thus selectively undergoing the thermal dehydration typical for aldoximes [22,23] with no signs of NMe_2_ group displacement. This experimental finding allowed us to compare the reaction profiles for reactive (towards the NMe_2_ group displacement) **1a** and inert **1b** in order to determine the most reasonable reaction mechanism and explain the difference in reactivity of these substrates. According to a NOESY experiment, aldoxime **1b**, similarly to **1a**, exists solely as an *E*-isomer (see Supplementary Materials). This allowed us to model the intermediates of the *E*-**1b** → **4b** transformation in a similar way as for *E*-**1a** → **4a** (Figure 2).

**Figure 3:**
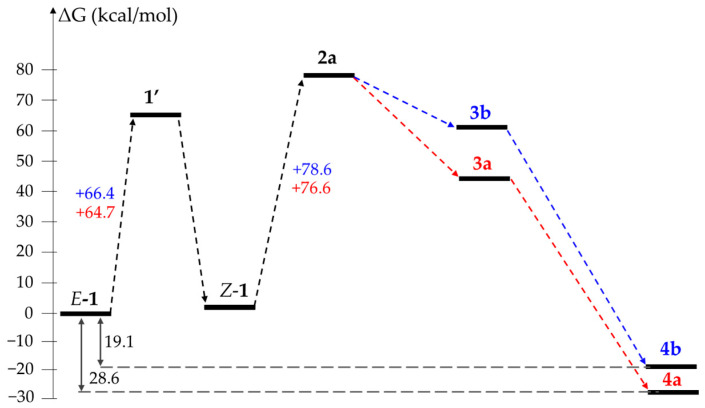
Comparison of reaction profiles for *E*-**1a** → **4a** (red) and *E*-**1b** → **4b** (blue) via “neutral” path A.

**Figure 4:**
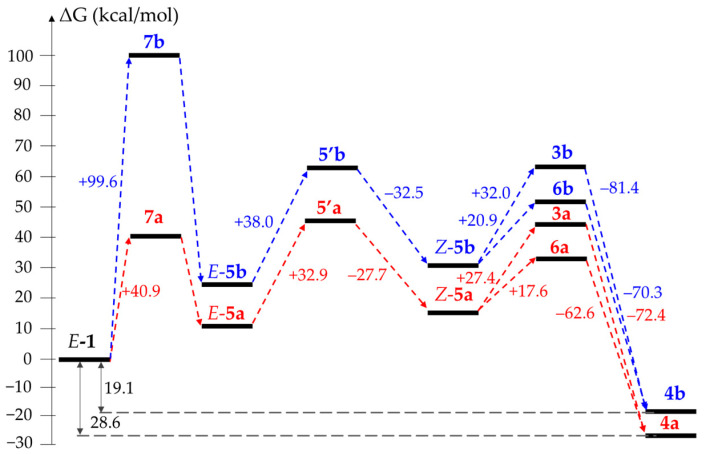
Comparison of reaction profiles for *E*-**1a** → **4a** (red) and *E*-**1b** → **4b** (blue) via “zwitterionic” path B.

**Figure 5:**
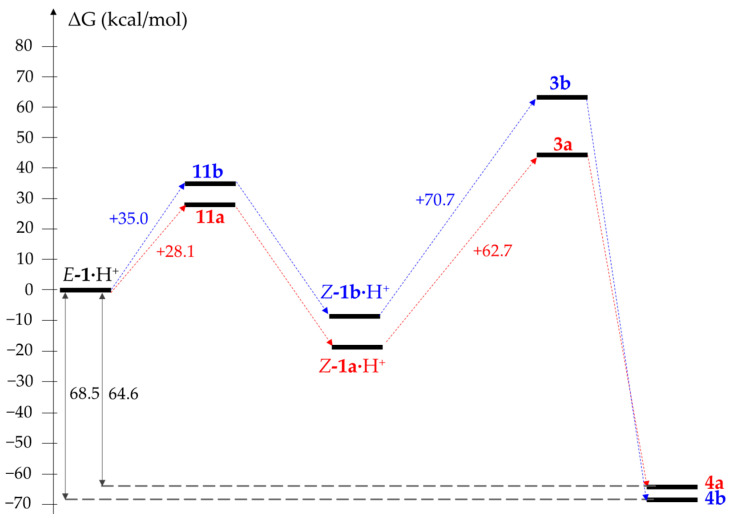
Comparison of reaction profiles for *E*-**1a**·H^+^ → **4a** (red) and *E*-**1b**·H^+^ → **4b** (blue) via “acidic” path C.

Based on the calculation of the free Gibbs energy difference between optimized structures, we have modeled the reaction profiles for the transformations **1a** → **4a** and **1b** → **4b** via the pathways A, B and C (Figure 3, Figure 4 and Figure 5). Since the dielectric constants of melted oximes **1a** and **1b** are unknown, all calculations were performed using DMSO as a solvent in the framework of a conductor-like polarizable continuum model (CPCM). It was found that regardless of the pathway, the process in total is thermodynamically favorable for both substrates: the energy gain for *E*-**1a** → **4a** and *E*-**1b** → **4b** is 28.6 and 19.1 kcal/mol, respectively. The formation of **1’a** (**1’b**) and **5’a** (**5’b**) requires 64.7 (66.4) and 32.9 (38.0) kcal/mol, respectively, which is in agreement with the activation barrier for the *E* → *Z* isomerization of aldoximes (~30–60 kcal/mol [24,25,26]). The protonation of both substrates significantly decreases *E* → *Z* isomerization barriers to 28.1 and 35.0 kcal/mol for **1a**·H^+^ and **1b**·H^+^, respectively.

The analysis of the “neutral” path A demonstrates almost no differences in the free Gibbs energy changes in stage *Z*-**1** → **2**—76.6 vs. 78.6 kcal/mol for **1a** and **1b**, respectively (Figure 3). However, further proton transfer reveals the higher stability of **3a** in comparison with **3b**. We attribute this difference to the stronger hydrogen bonding in **3a**.

The “zwitterionic” path B is characterized by the noticeable difference in the formation of intermediates **3**, **5**, **6** and (especially dramatically) **7** for the substrates **1a** and **1b** (Figure 4). Thus, the stage of proton transfer (**1** → **5**) is significantly facilitated for **1a**—the formation of transition states **7a** and **7b** requires 40.9 and 99.6 kcal/mol, respectively. We believe that this difference originated from the abnormally high basicity of the DMAN derivatives. At the same time, the transformation *Z*-**5a** → **3a** becomes noticeably more favorable than *Z*-**5b** → **6b** (27.4 vs. 32 kcal/mol) in contrast to *Z*-**1** → **2**. The concert ring closure and NMe_2_ group displacement appear preferable (≈10 kcal/mol) to the step-by-step process for both substrates, with the *Z*-**5a** → **6a** transition being slightly more facilitated than *Z*-**5b** → **6b** (17.6 vs. 20.9 kcal/mol).

**Scheme 7:**
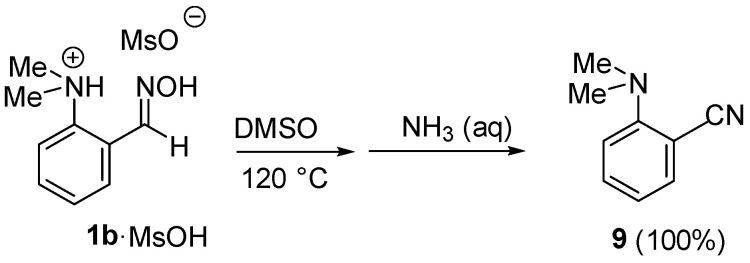
Thermolysis of oxime **1b**·MsOH.

The obtained data make it very tempting to conclude that the formation of transition state **7** is a limiting stage of the reaction. However, according to our experiments, the usage of protonated **1b**·MsOH and **8**·MsOH as starting materials still leads to the same outcome: after 24 h of heating in DMSO, **1b**·MsOH undergoes a simple dehydration into cyanide **9**, while ketoxime **8**·MsOH stays unchanged (Scheme 7). In contrast, it is known that the substitution of the NMe_2_ group in a proton sponge series still occurs (is even facilitated) in acidic media [12]. That brings us to the conclusion that the protonation of an NMe_2_ group indeed promotes the reaction, while the deprotonation of an oxime moiety is not mandatory. Indeed, the comparison of possible reaction profiles for the *E*-**1a**·H^+^ → **4a** and *E*-**1b**·H^+^ → **4b** transformations via the “acidic” path C reveals the major difference in free Gibbs energy at the stage when transition state **3** is formed—the transformation *Z*-**1a**·H^+^ → **3a** is significantly (62.7 vs. 70.0 kcal/mol) more favorable than *Z*-**1b**·H^+^ → **6b** (Figure 5).

**Figure 6:**
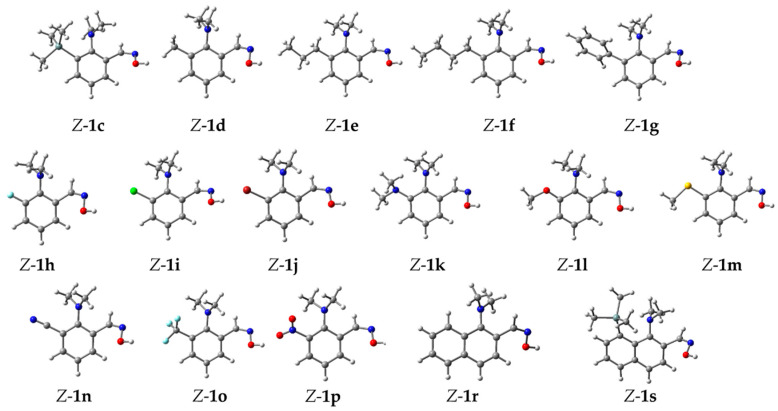
Optimized geometries of *Z*-aldoximes **1c**–**1s**.

Considering all the obtained data, we assume that the steric pressure of the 8-NMe_2_ group facilitates an intramolecular nucleophilic attack for all studied mechanisms A–C. Thus, the formation of intermediates **2, 3** and **6** is always to some extent preferable for sterically more strained **1a** than for **1b**. In order to prove this assumption, we performed calculations of the barriers for the formation of the intermediates **3**, **6** and **6**·H^+^ via all cyclization mechanisms A, B and C for a series of *N*,*N*-dimethylaniline based aldoximes (**1c**–**1p),** together with 1-dimethylaminonaphthalene-based aldoximes **1r** and **1s** (Figure 6). Indeed, the introduction of bulky substituents into the *ortho*-position (or *peri*-position) of the NMe_2_ group decreases the activation barrier for the transformations *Z*-**1** → **2** → **4**, *Z*-**5** → **3** (**6**) → **4** and *Z*-**1**·H^+^ → **3** (**6**) → **4** (Table 1). One can see that regardless of the mechanism, the tendencies in the changes of the activation barrier for the NMe_2_ group displacement are similar. It is noticeable that the NMe_2_ (**1k**) and OMe (**1l**) groups, despite their bulkiness, suppress an intramolecular nucleophilic attack due to their strong *+M*-effect destabilizing the negative charge in intermediate **3**. In contrast, the less bulky, strongly electron-withdrawing CF_3_ (**1o**), NO_2_ (**1p**) and CN (**1n**) groups significantly facilitate the reaction in comparison to **1a**. To our satisfaction, the DMA-based oximes **1c** and **1s**, containing bulky SiMe_3_ substituents, generally have the lowest barriers for the NMe_2_ substitution stage for all studied mechanisms.

Even though the calculated barriers for substrates that are reactive and unreactive towards the NMe_2_ substitution do not dramatically differ, the tendency in their change appears to be very convincing, depending on the introduced substituent.

**Figure 7:**
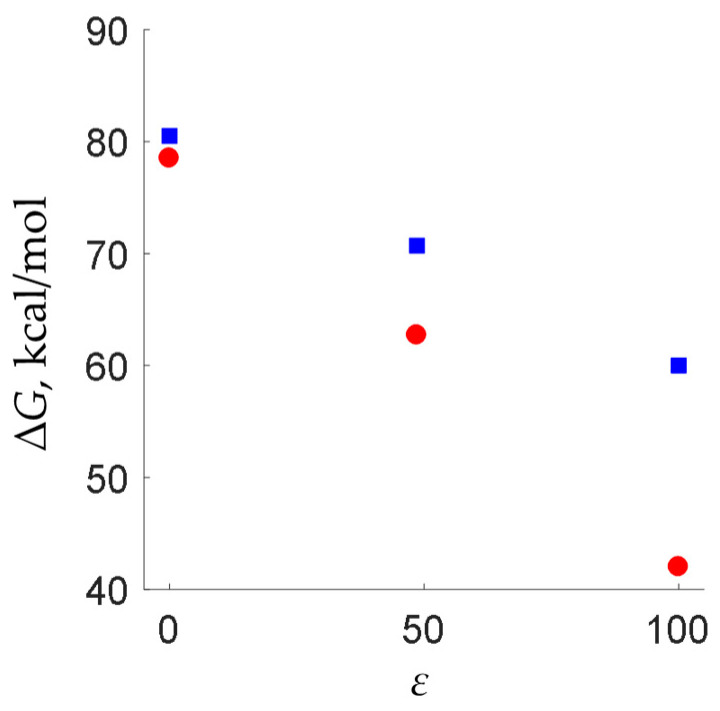
Activation barriers for *Z*-**1a**·H^+^→**3a** (red circles) and *Z*-**1b**·H^+^→**3b** (blue squares) calculated with *ε* = 1 (vacuum), *ε* = 48.6 (dimethyl sulfoxide) and *ε* = 100.

It should be specially noted that we used the conductor-like polarizable continuum model (CPCM) with dimethylsulfoxide as a solvent in order to take non-specific solvation effects into account. Test calculations for the *Z*-**1**·H^+^→**3** activation barrier in vacuum, in DMSO and in artificial solvent with rather high dielectric permittivity (ε = 100) show that the ∆*G* values vary significantly (Figure 7). However, the overall tendency remains the same—for oxime **1b**, the activation barrier is higher than that for sterically strained **1a**. Thus, the results of the calculations of activation barriers should be mostly perceived in a qualitative sense. In order to obtain more dramatic differences in barriers (as was expected from the experimental data), one should also take specific solvent effects (solvent–solute interactions) into account, as well as the macroscopic reaction field, which, of course, involves additional computational cost [27].

Thus, we expect that the reactivity of sterically strained oximes **1c** and **1s** will be comparable (if not superior) to **1a** in real experimental conditions. Keeping in mind that SiMe_3_ can be easily introduced to the NMe_2_ group in the *ortho*- or *peri*-position via organometallic reagents [28], we believe that the presented findings will open up a simple way for the synthesis of novel polynuclear isoxazoles.

## 3. Materials and Methods

The geometry optimization and calculations of vibrational harmonic frequencies were performed using the Gaussian16 software package [29]. Optimized geometries were calculated at the ωB97xd/def-2-TZVP level of theory. The chosen functional and basis set provide a reasonable combination of accuracy and computing efficiency in the calculations of both the geometries and the thermodynamic parameters [30]. The calculations were performed using the Polarizable Continuum Model (CPCM) and dimethyl sulfoxide as a solvent.

All the obtained structures were verified via their correspondence to a real local minimum by checking the absence of imaginary frequencies. Transition states were located as maxima points on 1D relaxed scans along the reaction coordinate. Saddle points were optimized using the Berny algorithm and verified by frequency calculations (i.e., existence of a single imaginary frequency).

Free Gibbs energies were estimated by virtue of thermochemistry data obtained from vibrational harmonic frequencies calculations. Thermochemical values were calculated at 400 K and atmospheric pressure (the reaction conditions for compound **1a**) in order to obtain the appropriate activation energies of the investigated processes. The change in free Gibbs energy was calculated as the difference between the two forms of interest while taking possible reagents and products into account.

Liquid-state NMR experiments were performed at the Centre for Magnetic Resonance, St. Petersburg State University. Chemical shifts are referred to as TMS.

### 3.1. Synthesis of ***1b***

As was previously described, *N*,*N*-dimethylaniline (1 mL, 7.9 mmol), freshly distilled over sodium-benzophenone hexane (15.0 mL), freshly distilled over KOH TMEDA (0.18 mL, 1.185 mmol), and a 1.6 M solution of *n*-BuLi in hexanes (5 mL, 8 mmol) were added to a dry round-bottom flask equipped with a stirring bar under argon atmosphere. The solution was stirred at 72 °C for 48 h and cooled to −20 °C. Dry DMF (0.75 mL, 9.25 mmol) was added to the reaction mixture via syringe. The reaction mixture was warmed up to room temperature, and stirred for 4 h, then treated with water (50 mL). The products were extracted with diethyl ether (3 × 50 mL), the solvent was evaporated to dryness and the residue was chromatographed on alumina with an EtOAc/petroleum ether 1:15 mixture as the eluent. A yellow fraction with R_f_ = 0.7 was collected to yield 940 mg (6.3 mmol, 80%) of 2-(Dimethylamino)benzaldehyde as yellow oil. The characterization data were consistent with those reported in the literature [31]. δ_H_(400 MHz, Chloroform-*d*): 10.25 (1H, s), 7.78 (1H, dd, *J* = 7.7, 1.8 Hz), 7.48 (1H, ddd, *J* = 8.7, 7.1, 1.8 Hz), 7.07 (1H, dd, *J* = 8.4, 1.0 Hz), 7.02 (1H, t, *J* = 7.5 Hz), 2.94 (6H, s).

A solution of hydroxylamine hydrochloride (420 mg, 6 mmol) in 10 mL of ethanol was added to the solution of 2-(Dimethylamino)benzaldehyde (600 mg, 4 mmol) in 20 mL of ethanol. The reaction mixture was stirred under reflux for 2 h. The solvent was removed in vacuum and the resulting white powder was mixed with 20 mL of aq. ammonia and 20 mL of dichloromethane. The organic layer was separated, and the water layer was extracted with dichloromethane (3 × 20 mL). The combined organic fraction was dried over sodium sulphate. The solvent was removed, and the residue was recrystallized from hexane, yielding 837 mg (5.1 mmol, 85%) of 2-Dimethylamino-benzaldehyde-oxime as colorless needle crystals. δ_H_(400 MHz, Chloroform-*d*): 8.90 (1H, s), 8.50 (1H, s), 7.70 (1H, d, *J* = 7.6 Hz), 7.36 (1H, t, *J* = 7.6 Hz), 7.10 (1H, d, *J* = 8.2 Hz), 7.06 (1H, t, *J* = 7.5 Hz), 2.78 (6H, s). δ_c_(100 MHz, Chloroform-*d*): 153.07, 148.99, 130.60, 127.69, 125.39, 122.67, 118.56, 45.20

### 3.2. Thermolysis of ***1b***

2-Dimethylamino-benzaldehyde-oxime (100 mg, 0.61 mmol) was placed in a test tube and heated at 140 °C for 84 h. The residue was dissolved in 0.6 mL of CDCl_3_ and analyzed by NMR spectroscopy to reveal cyanide **9** as a single product. The characterization data were consistent with those reported in the literature [31].

### 3.3. Thermolysis of ***1b***·MsOH

2-Dimethylaminobenzaldehyde oxime **1b** (300 mg, 1.83 mmol) was placed in a sample tube and dissolved in 3 mL of diethyl ether. Then, 180 mg (1.88 mmol) of methanesulfonic acid dissolved in 3 mL of diethyl ether were added. The resulting suspension was centrifuged, and the precipitate was washed with diethyl ether to yield 476 mg (1.83 mmol, 100%) of **1b**·MsOH as colorless crystals. δ_H_(400 MHz, DMSO-*d*_6_): 12.07 (1H, s), 8.98 (1H, s), 8.43 (1H, s), 7.85 (1H, d, *J* = 7.7 Hz), 7.72 (1H, d, *J* = 7.6 Hz), 7.58 (1H, t, *J* = 7.4 Hz), 7.49 (1H, t, *J* = 7.4 Hz), 3.16 (6H, s), 2.46 (3H, s).

Then, 350 mg (1.34 mmol) of 2-Dimethylamino-benzaldehyde-oxime methanesulfonate were placed in a sample tube, dissolved in 3 mL of DMSO-d_6_ and heated at 120 °C for 24 h. The reaction mixture was cooled to room temperature and mixed with 20 mL of aq. ammonia and 20 mL of dichloromethane. The organic layer was separated, and the water layer was extracted with dichloromethane (3 × 20 mL). The combined organic fraction was dried over sodium sulphate. The solvent was evaporated to dryness and the residue was chromatographed on an alumina with EtOAc-petroleum ether 1:10 mixture as eluent. The pale yellow fraction with R_f_ = 0.9 was collected to yield **9** as a pale yellow oil. The characterization data were consistent with those reported in the literature [31].

## 4. Conclusions

Based on the obtained data, we can conclude that the steric pressure of the 8-NMe_2_ group facilitates an intramolecular nucleophilic attack to C-1 carbon and serves as the major source of reactivity for proton sponge *ortho*-oximes, while the protonation of the NMe_2_ group serves as a supporting factor. We have demonstrated that a similar (and even lower) activation barrier can be achieved in a dimethylaniline and dimethylaminonaphthalene series via the introduction of bulky substituents to the *ortho*- or *peri*-position of NMe_2_ groups. Better reactivity is expected from substrates containing trimethylsilyl groups, while the introduction of electron-withdrawing CF_3_, NO_2_ and CN groups is less effective. The combination of NMe_2_ group protonation with the steric pressure of the bulky substituent appears to be the most effective for the facile NMe_2_ group’s displacement. The predicted reactivity could serve as a simple approach to the synthesis of fused isoxazoles from sterically strained *ortho*-oximes of dimethylaniline and dimethylaminonaphthalene.

## Figures and Tables

**Table 1:**
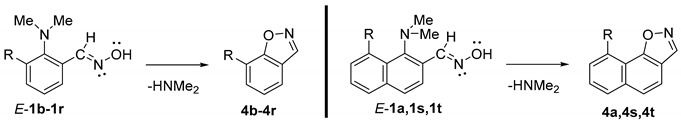
Calculated activation barriers for the transformation of aldoximes **1a**–**1s** into isoxazoles **4a**–**4s**.

Starting Material	R	Activation Barriers, kcal/mol				
		*Z*-1 → 2	*Z*-5 → 3	*Z*-5 → 6	*Z*-1·H^+^ → 3	*Z*-1·H^+^ → 6
**1a**	NMe_2_	76.7	27.4	17.6	62.7	52.9
**1b**	H	78.6	32.0	20.7	70.7	59.4
**1c**	SiMe_3_	72.6	23.5	19.8	56.9	53.2
**1d**	Me	78.9	32.0	22.9	67.6	58.5
**1e**	*i*Pr	78.4	31.1	23.0	66.6	58.5
**1f**	*t*Bu	78.3	32.0	23.1	67.0	58.1
**1g**	Ph	80.1	33.9	23.2	67.8	57.2
**1h**	F	83.4	36.5	21.9	69.6	54.9
**1i**	Cl	78.6	31.4	21.5	64.2	54.3
**1j**	Br	77.2	29.5	20.6	62.3	53.4
**1k**	NMe_2_	82.2	41.5	23.7	75.5	57.7
**1l**	OMe	80.2	40.9	25.6	75.1	59.9
**1m**	SMe	83.4	31.1	17.4	62.3	51.6
**1n**	CN	86.0	23.7	13.7	55.4	45.4
**1o**	CF_3_	85.8	22.8	17.9	56.0	51.2
**1p**	NO_2_	83.8	24.3	14.4	55.7	45.8
**1r**	H	77.2	36.4	15.6	70.0	49.2
**1s**	SiMe_3_	72.3	8.7	7.7	45.3	44.3

## Supplementary Materials

The following are available online, Figure S1: ^1^H NMR spectrum of oxime **1b** (CDCl_3_, 400 MHz), Figure S2: ^13^C NMR spectrum of oxime **1b** (CDCl_3_, 100 MHz), Figure S3: ^1^H-^1^H NOESY spectrum of oxime **1b** (CDCl_3_, 400 MHz), Figure S4: ^1^H NMR spectrum of oxime **1b**·MsOH (DMSO-d_6_, 400 MHz).
