# Supplementary material for: Sterically Facilitated Intramolecular Nucleophilic NMe2 Group Substitution in the Synthesis of Fused Isoxazoles: Theoretical Study
Source: Molecules. 2020 Dec 17;25(24):5977. doi: 10.3390/molecules25245977 (PMC7765840; doi:10.3390/molecules25245977)
Supplement: Supplementary file 1 [file molecules-25-05977-s001.pdf]

Supporting Information for

Sterically facilitated intramolecular nucleophilic NMe_2 group substitution in the synthesis of fused isoxazoles: theoretical study

Alexander S. Antonov,* Elena Yu. Tupikina, Valerii V. Karpov, Valeriia V. Mulloyarova and Victor G. Bardakov

[1] Institute of Chemistry, St. Petersburg State University, Universitetskii pr. 26, 198504 St. Petersburg, Russian Federation
* Correspondence: Aleksandr.antonov@spbu.ru

Copies of NMR spectra of obtained compounds……………………………………………………….S-2

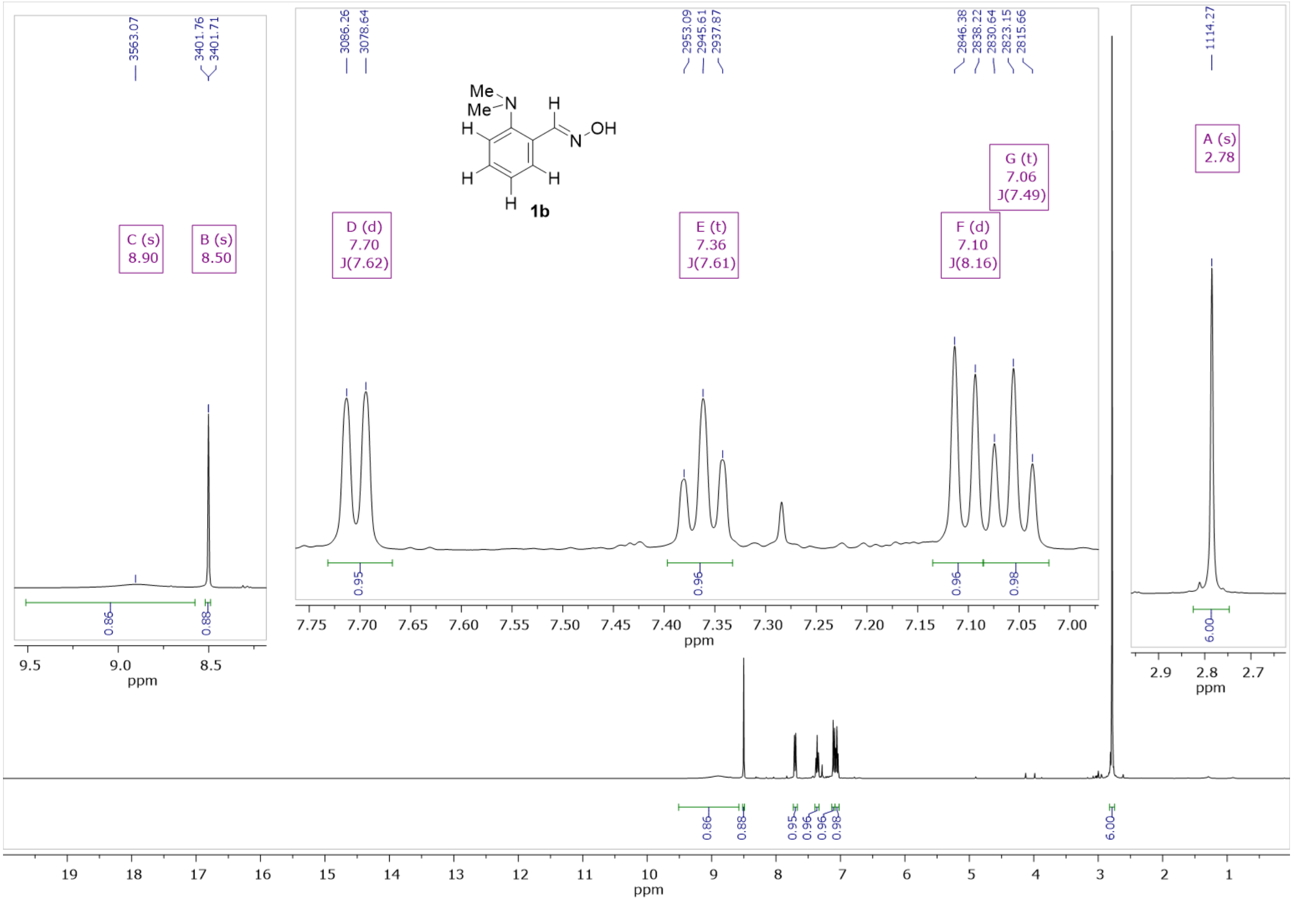

Figure S1: ^{1}H NMR spectrum of oxime **1b** ($CDCl_3$, 400 MHz)

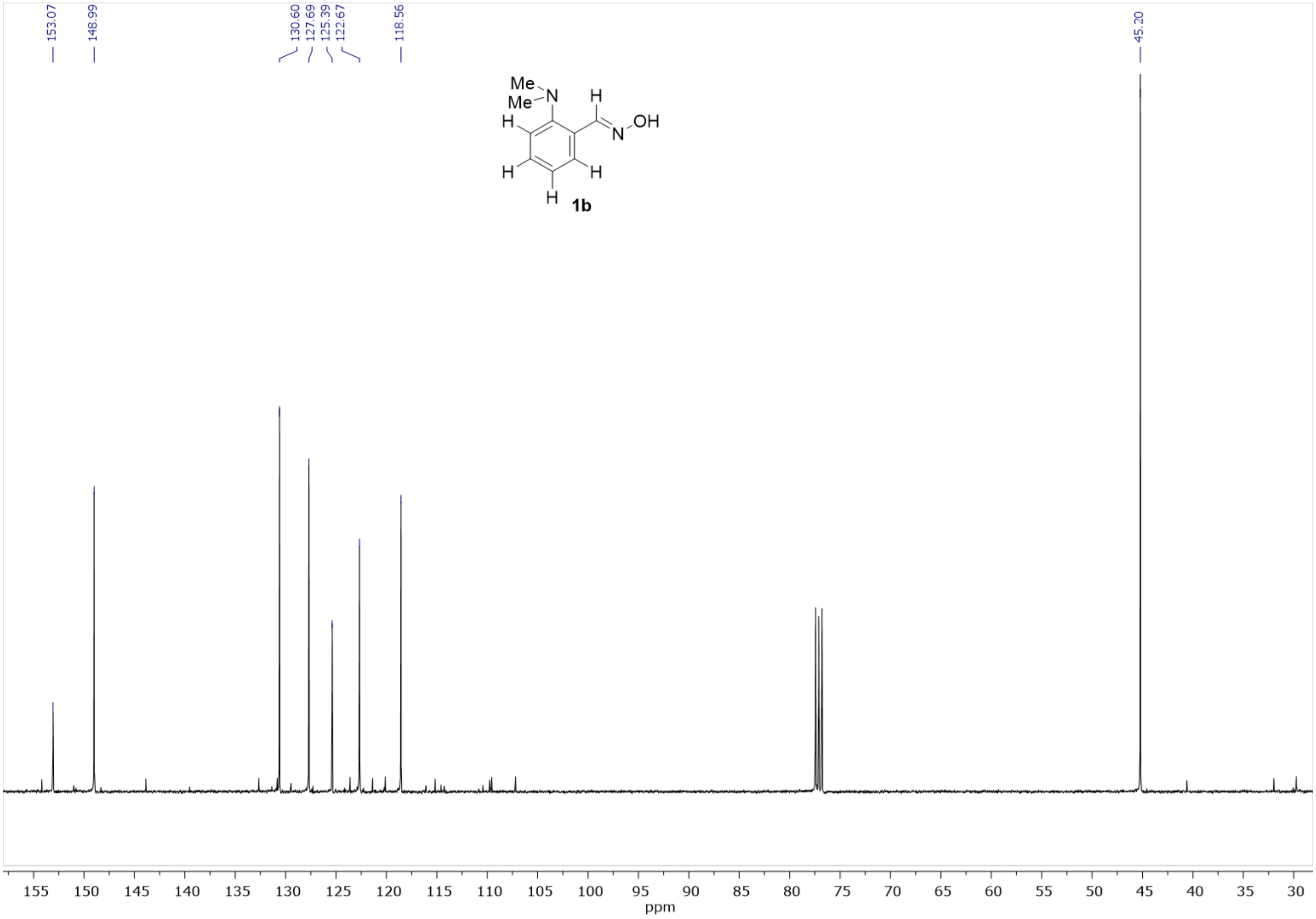

Figure S2: ^{13}C NMR spectrum of oxime **1b** ($CDCl_3$, 100 MHz)

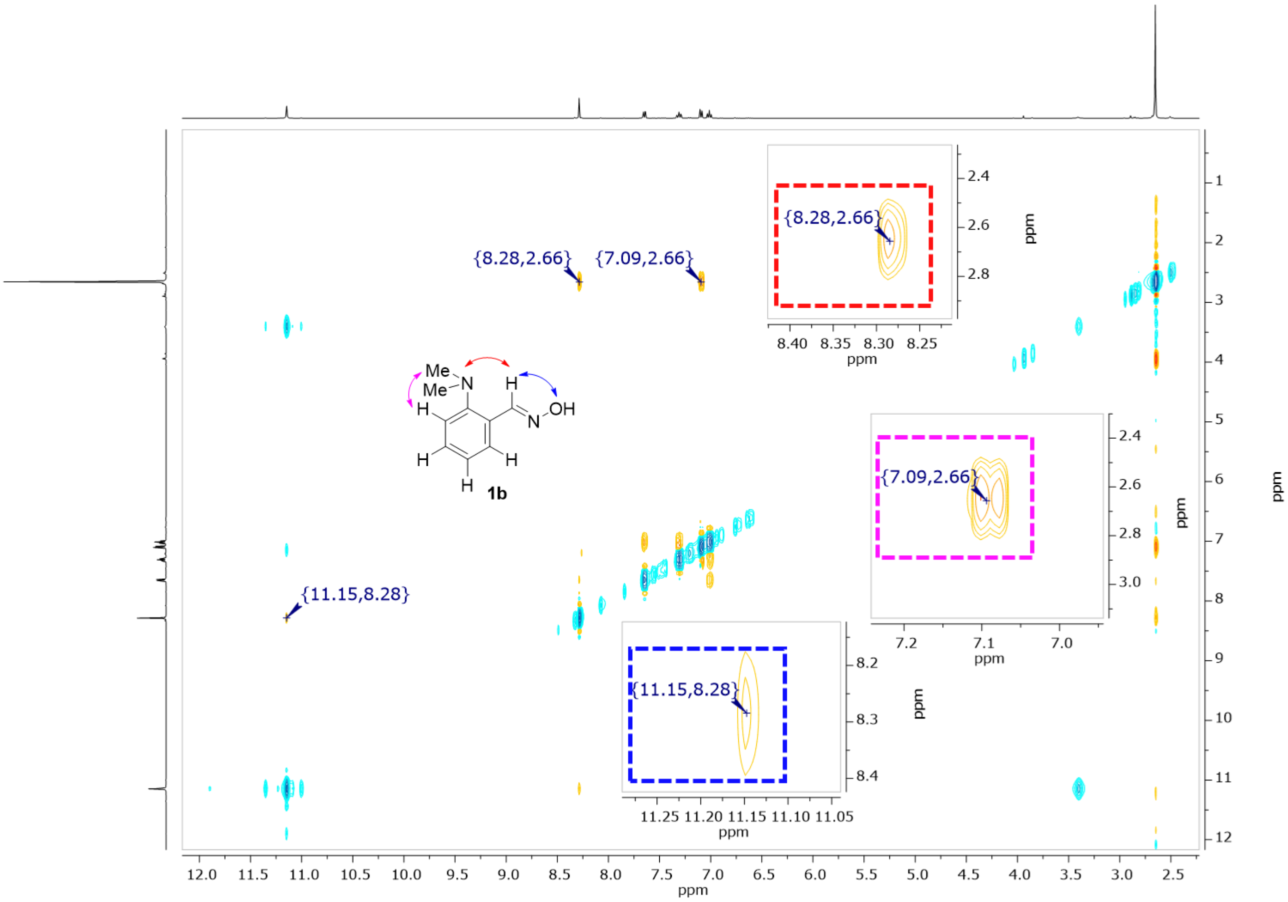

Figure S3: ^{1}H-^{1}H NOESY spectrum of oxime **1b** ($CDCl_3$, 400 MHz)

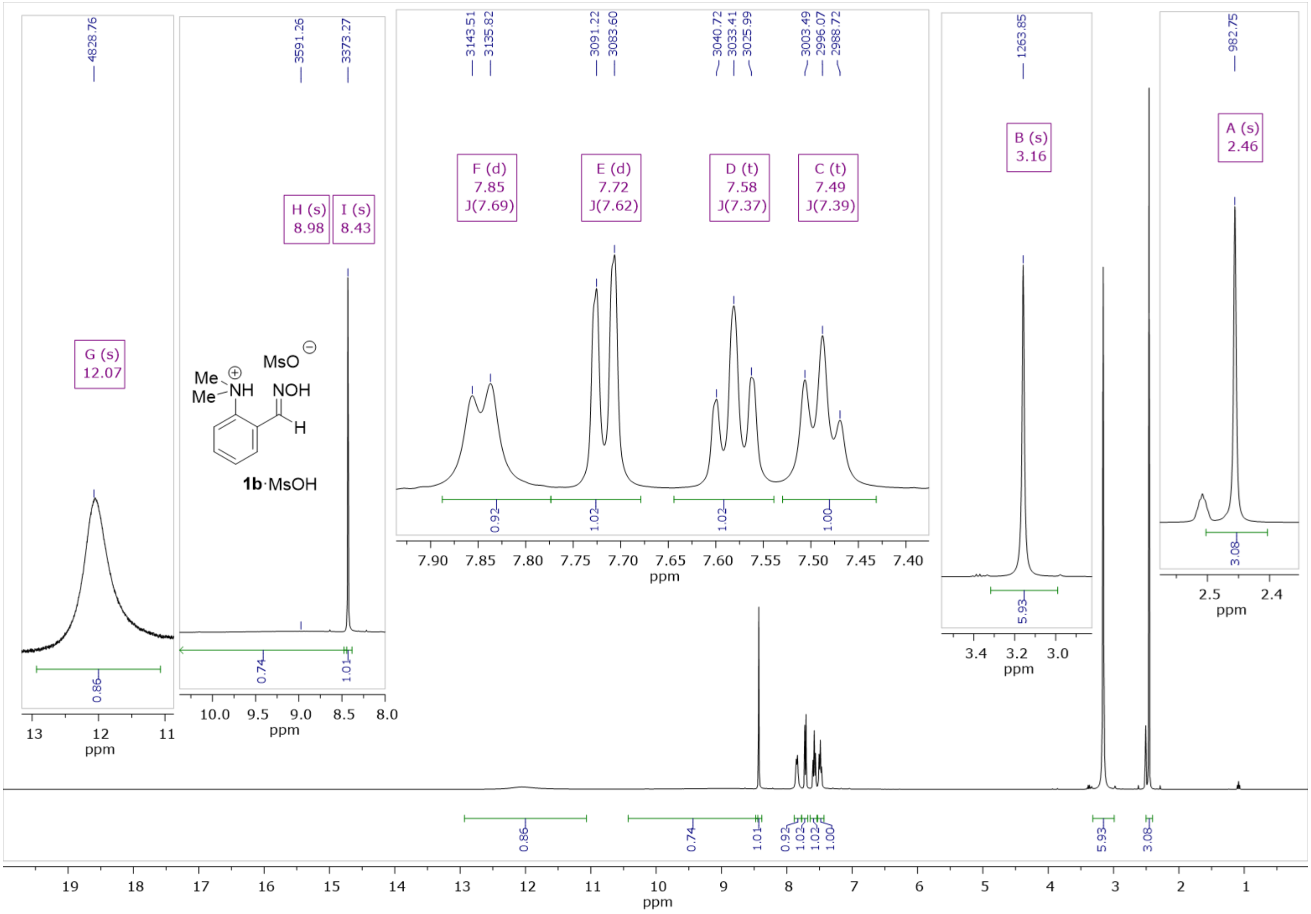

Figure S4: ^{1}H NMR spectrum of oxime **1b**·MsOH (DMSO-d_6, 400 MHz),
